# Validation of a self-reported HIV symptoms list: the ISS-HIV symptoms scale

**DOI:** 10.1186/s12981-016-0102-2

**Published:** 2016-04-09

**Authors:** Raffaella Bucciardini, Katherina Pugliese, Daniela Francisci, Andrea Costantini, Elisabetta Schiaroli, Miriam Cognigni, Chiara Tontini, Stefano Lucattini, Luca Fucili, Massimiliano Di Gregorio, Marco Mirra, Vincenzo Fragola, Sara Pompili, Rita Murri, Stefano Vella

**Affiliations:** Istituto Superiore di Sanità, Rome, Italy; Ospedale S. Maria della Misericordia, Perugia, Italy; Azienda Ospedaliera Universitaria Ospedali Riuniti, Ancona, Italy; University of Rome “La Sapienza”, Rome, Italy; Institute of Infectious Diseases, Catholic University of Rome, Rome, Italy

**Keywords:** Patients reported outcomes, Health related quality of life, HIV-symptoms list

## Abstract

**Background:**

To describe the development and the psychometric properties of the Istituto Superiore di Sanità-HIV symptoms scale (lSS-HIV symptoms scale).

**Methods:**

The ISS-HIV symptom scale was developed by an Italian working team including researchers, physicians and people living with HIV. The development process went through the following steps: (1) review of HIV/AIDS literature; (2) focus group; (3) pre-test analysis; (4) scale validation.

**Results:**

The 22 symptoms of HIV-ISS symptoms scale were clustered in five factors: pain/general discomfort (7 items); depression/anxiety (4 items); emotional reaction/psychological distress (5 items); gastrointestinal discomfort (4 items); sexual discomfort (2 items). The internal consistence reliability was for all factors within the minimum accepted standard of 0.70.

**Conclusions:**

The results of this study provide a preliminary evidence of the reliability and validity of the ISS-HIV symptoms scale. In the new era where HIV infection has been transformed into a chronic diseases and patients are experiencing a complex range of symptoms, the ISS-HIV symptoms scale may represent an useful tool for a comprehensive symptom assessment with the advantage of being easy to fill out by patients and potentially attractive to physicians mainly because it is easy to understand and requires short time to interpret the results.

## Background

The physician’s assessment was for a long time the only factor that affected health care decisions, however in more recent years the necessity for a closer cooperation between physician and patient has been recognized. In this contest a key role is represented by the patient reported outcomes (PROs). Food and Drug Administration (FDA) defined PROs as any report coming directly from patients about their health condition and treatment [1]. PROs can be used to measure overall concepts (e.g. state of general health), presence and intensity of specific symptoms, complex concepts, like the health related quality of life (HRQoL) [2–11]. In the field of HIV disease, symptoms play a central role in the life of HIV patients: they are the first reason the patient asks for care; they are strongly associated with HRQoL; they represent the patients experience of the drug adverse effects and disease effects [12–16]. Effective symptom management, aimed to decrease the burden of symptoms, could have a direct impact on improving the HRQoL and also an indirect impact on improving the adherence to antiretroviral therapy (ART) with expected positive impact on the efficacy of the treatment [17–20]. Moreover the clinical interpretation of the symptoms is easily understood by physicians with a likely immediate effect in clinical practice [21]. It has also been shown that the symptoms reported directly by patients are more complete and more strongly associated with HRQoL than provider-reported symptoms. [6]. It is therefore necessary that the presence and impact of symptoms are reported directly by the patient through the use of specific tools. An HIV specific symptom checklist might be the most suitable tool for routine clinical use [21]. In the present paper we describe the development and the psychometric properties of the Istituto Superiore di Sanità-HIV symptoms scale (lSS-HIV symptoms scale).

## Methods

The ISS-HIV symptom scale was developed by an Italian working team (coordinated by the Italian National Institute of Health, the ISS), including researchers, physicians and people living with HIV. The development process went through the following steps:

### 1. Review of existing HIV/AIDS literature

The first step in the development of the ISS-HIV symptoms scale was to review the symptoms measures available in literature. As a result of regular meeting the working team identified a list of symptoms to be included in the instrument.

### 2. Focus group

Two focus groups were conducted on independent groups of approximately 10 HIV infected people. The patients were recruited both in clinical centers and patients’ associations. The work done by focus groups resulted in a first draft of 38 HIV-related symptoms including.

### 3. Pre-test analysis

The first draft of the symptoms list was tested in a cross-sectional study on a group of 200 HIV + people, taking ART, recruited in two Italian clinical centers (Azienda Ospedaliera Universitaria Ospedali Riuniti, Ancona; Ospedale S. Maria della Misericordia, Perugia). Frequency analysis of symptoms and an exploratory principal components factor analysis were conducted. Based on these results, the preliminary item list has then been modified. Twenty-two of the 38 original symptoms were retained for the final version of the symptoms list.

### 4. HIV-ISS symptoms scale validation and final structure

A sample of 161 HIV + people on ART, enrolled in a cross-sectional study, from the two Italian clinical centers (Azienda Ospedaliera Universitaria Ospedali Riuniti, Ancona; Ospedale S. Maria della Misericordia, Perugia), was used to carry out the validation analysis. This study received ethics approval, and all patients provided written informed consent (*name of the Ethical Committee: Istituto Superiore di Sanità, Ethical Review Board*). The sample was defined on the basis of common practice in psychometric studies, according to which at least five cases for each item should be provided. In this study the case-to-item ration was approximately 7.3. The final version of HIV-ISS symptoms scale resulted in a self- administered list of 22 symptoms. A Likert five-point intensity scale was used to evaluate the impact of symptoms (Appendix).

## Statistical analysis

The statistical analyses reported in this study refer to validation analysis of HIV-ISS symptom scale including psychometric assumptions of validity and reliability [22]. An instrument is said to be valid if it measures what it is supposed to measure. For this purpose we focused on the concurrent validity and construct validity. The construct validity of the HIV-ISS symptoms scale was assessed using principal components factor analysis with varimax rotation. As for concurrent validity, a step-wise multiple regressions were used to examine the relationship between the HIV-ISS symptoms scale and an external and independent measure of HRQoL. For this purpose the ISSQoL (Istituto Superiore di Sanità-Quality of Life), a validated specific questionnaire designed for people living with HIV, was used [23, 24]. The ISSQoL questionnaire includes 9 domains: physical functioning, role functioning, depression/anxiety, energy/vitality, health distress, cognitive functioning, social functioning, sexual life and satisfaction with quality of life. Reliability is defined as the degree to which repeated administration of a measurement instrument produces equivalent results under controlled conditions. In the present study the Cronbach’s α coiffecient was used as an estimate of the test reliability. The range between 0.70 and 0.90 is considered as the minimum required value.

## Results—validation analysis

### Description of the sample

The characteristics of the sample used for the validation analysis are shown in Table 1. The majority were men (75.8 %); 42.9 % were heterosexual. Median age was 49 years old. About more than half of the participants were asymptomatic (56.5 %) and they were HIV infected for a median of 13.5 years. Median HIV viral load was 1.6 log and median CD4 cell count was 611 cells/mm^3^.

**Table 1:** Characteristics of the population used for the validation analysis

Sex n (%)	
Female	39 (24.2)
Male	122 (75.8)
Age (years)	
Mean ± SD (n, range)	49.0 ± 9.8 (161, 25–77)
Median	49.0
Transmission route, n (%)	
Men having sex with men	60 (37.3)
Intravenous drug users	26 (16.1)
Heterosexual	69 (42.9)
Other	6 (3.7)
HIV status, n (%)	
Asymptomatic (CDC A)	91 (56.5)
Symptomatic (CDC B	47 (29.2)
AIDS (CDC C)	23 (14.3)
Time from HIV diagnosis (years)	
Mean ± SD (n, range)	13.4 ± 9.2 (160, 0–30)
Median	13.5
CD4 (cells/mm^3^)	
Mean ± SD (n, range)	652 ± 312 (161, 24–1686)
Median	611
HIV-RNA cp/ml (log_10_)	
Mean ± SD (n, range)	1.76 ± 0.6 (161, 1–6)
Median	1.60

### Construct validity

The 22 symptoms of HIV-ISS symptoms scale were clustered in five factors that explained 64.96 % of variance (Table 2): pain/general discomfort (7 items);depression/anxiety (4 items); emotional reaction/psychological distress (5 items); gastrointestinal discomfort (4 items);sexual discomfort (2 items);

**Table 2:** Factor structure and reliability estimates

ISS-HIV symptom scales Items	Loadings	% Variance	Chronbach’s α
*Factor 1: Pain/general discomfort (7 items)*		16.25	0.86
Pain in the head or feet	0.769		
Aching muscles	0.691		
Abnormal accumulation of fat	0.681		
Abdominal bloating	0.563		
Sweating	0.513		
Impaired vision	0.498		
Headache	0.475		
*Factor 2: Depression, anxiety (4 items*)		16.03	0.87
Feeling sad	0.817		
Worrying	0.758		
Feeling anxious and nervous	0.718		
Sleep problems	0.574		
*Factor 3: Emotional reaction/psychological distress (5 items)*		13.08	0.83
Memory problems	0.739		
Coughing	0.692		
Shortness of breath	0.608		
Difficulty in concentrating	0.591		
Tiredness	0.488		
*Factor 4: Gastrointestinal discomfort (4 items)*		10.02	0.70
Weight loss	0.715		
Lack of appetite	0.702		
Diarrhoea	0.656		
Heartburn	0.508		
*Factor 5: Sexual discomfort (2 items)*		9.58	0.91
Decrease of sexual interest	0.790		
Problem with sexual activity	0.758		

### Reliability

The Chronbach’s α scores were for all factors within the minimum accepted standard of 0.70 (Table 2).

### Concurrent validity

Step-wise multiple regressions were used for each ISSQoL domain as dependent variable and the five factors of the HIV-ISS symptoms scale as predictor variables. The HIV-ISS symptoms scale concurrent validity was tested based on the following hypothesized significant relationships: (1) pain/general discomfort with physical functioning, social functioning, depression/anxiety, energy/vitality, sexual life and satisfaction with quality of life; (2) depression/anxiety with all ISSQoL domains; (3) emotional reaction/psychological distress with social functioning, depression/anxiety, energy/vitality, cognitive functioning, satisfaction with quality of life; (4) gastrointestinal discomfort with social functioning, depression/anxiety, energy/vitality; (5) sexual discomfort with social functioning, depression/anxiety, sexual life, satisfaction with quality of life. All hypothesized relationships were confirmed (Table 3).

**Table 3:** Regression analysis between ISS-HIV factors scores and ISS-Qol questionnaire

**ISSQoL domains (dependent variables)**	**ISS-HIV factors score (predictors)**	Standardized coefficients (Beta)	p
Physical functioning	Factor 1—Pain/general discomfort	0.25	0.001
	Factor 2—Depression, anxiety	0.23	0.001
	Factor 3—Emotional reaction/psychological distress	0.16	0.030
	Factor 4—Gastrointestinal discomfort	0.13	0.068
	Factor 5—Sexual discomfort	0.29	0.000
Role functioning	Factor 1—Pain/general discomfort	−0.02	0.827
	Factor 2—Depression, anxiety	0.23	0.005
	Factor 3—Emotional reaction/psychological distress	0.17	0.033
	Factor 4—Gastrointestinal discomfort	0.09	0.262
	Factor 5—Sexual discomfort	0.17	0.039
Social functioning	Factor 1—Pain/general discomfort	0.22	0.001
	Factor 2—Depression, anxiety	0.42	0.000
	Factor 3—Emotional reaction/psychological distress	0.19	0.004
	Factor 4—Gastrointestinal discomfort	0.19	0.005
	Factor 5—Sexual discomfort	0.22	0.001
Depression/anxiety	Factor 1—Pain/general discomfort	0.33	0.000
	Factor 2—Depression, anxiety	0.65	0.000
	Factor 3—Emotional reaction/psychological distress	0.26	0.000
	Factor 4—Gastrointestinal discomfort	0.23	0.000
	Factor 5—Sexual discomfort	0.27	0.000
Energy/vitality	Factor 1—Pain/general discomfort	0.19	0.002
	Factor 2—Depression, anxiety	0.40	0.000
	Factor 3—Emotional reaction/psychological distress	0.41	0.000
	Factor 4—Gastrointestinal discomfort	0.16	0.009
	Factor 5—Sexual discomfort	0.13	0.032
Health distress	Factor 1—Pain/general discomfort	0.22	0.000
	Factor 2—Depression, anxiety	0.58	0.000
	Factor 3—Emotional reaction/psychological distress	0.06	0.353
	Factor 4—Gastrointestinal discomfort	0.17	0.006
	Factor 5—Sexual discomfort	0.23	0.000
Cognitive functioning	Factor 1—Pain/general discomfort	0.19	0.001
	Factor 2—Depression, anxiety	0.46	0.000
	Factor 3—Emotional reaction/psychological distress	0.39	0.000
	Factor 4—Gastrointestinal discomfort	0.17	0.004
	Factor 5—Sexual discomfort	0.19	0.001
Sexual life	Factor 1—Pain/general discomfort	0.22	0.002
	Factor 2—Depression, anxiety	0.35	0.000
	Factor 3—Emotional reaction/psychological distress	0.03	0.623
	Factor 4—Gastrointestinal discomfort	−0.05	0.459
	Factor 5—Sexual discomfort	0.39	0.000
Satisfaction with quality of life	Factor 1—Pain/general discomfort	0.29	0.000
	Factor 2—Depression, anxiety	0.46	0.000
	Factor 3—Emotional reaction/psychological distress	0.14	0.033
	Factor 4—Gastrointestinal discomfort	0.07	0.285
	Factor 5—Sexual discomfort	0.19	0.003

## Discussion

The results of this study provide a preliminary evidence of the reliability and validity of the 22-item ISS-HIV symptoms scale. Factor analysis showed a good evidence for construct validity and internal consistency reliability. A good concurrent validity was also demonstrated. The regression analysis supported the evidence for the concurrent validity of the ISS-HIV symptoms scale factors with the ISSQoL domains. In the new era where HIV infection has been transformed into a chronic diseases and patients are experiencing a complex range of symptoms due to HIV infection, side effects of antiretroviral therapy (ART) and also associated comorbidities, the ISS-HIV symptoms scale may represent an updated and useful tool for a comprehensive symptom assessment and control in clinical practice.

Routine use of the symptoms assessment in the clinical practice could improve the communication between patient and clinician and this would make it possible to take targeted actions to manage the presence of particular symptom clusters [3, 25]. The use of patient-reported symptoms in clinical practice could not only improve clinician-patient communication, but also would allow to understand the changes in patient well-being over time, leading clinicians to therapeutic choices targeted to individual patients with a positive effect on individual patient management and outcomes [26–29]. The ISS-HIV symptoms scale has the advantage of being easy to fill out by patients and potentially attractive to physicians mainly because it is easy to understand and requires short time to interpret the results. The ISS-HIV Symptom Scale can be suitable for routine use in clinical practice, both as a single screening tool and in conjunction with other specific instruments. The simultaneous use of multiple instruments can be strategically significant in the presence of high-impact specific symptoms. The ISS-HIV Symptom Scale is able to detect the most frequent symptoms, as well as symptoms that may highly affect the patient’s quality of life. More specific instruments, such as those for pain or anxiety detection, could then be used to achieve more accurate information.

Considering also the recent agenda by the Patient-Centered Outcomes Research Institute (PCORI) that calls for a greater involvement of patients in assessing health care options, the systematic collection of PROs should be widely recommended [30]. As some experts in outcomes research have already experienced, a website, where PRO measures are collected in electronic health records (EHR) and linked to electronic medical record (EMR), can be a useful innovative tool to improve and stimulate the use of patient reported data in clinical practice [31, 32]. Clinicians would have the advantage to use PROs measure as a part of patients information along with laboratory and clinical data. The HIV-ISS symptoms scale, a short and comphrensive list of symptoms and easy to administer both by patients and clinicians, may be suitable for this purpose. The present study has several limitations. First, the construct validity of HIV-ISS Symptoms scale has not been supported by a confirmatory factor analysis. Moreover, a responsiveness assessment of the instrument would also be necessary. Generalization of results is limited to the Italian context where the validation analysis has been performed. Additional studies should address the reproducibility of index symptoms.

## Conclusions

The results of this study show that ISS-HIV symptoms scale is a self-administered tool, with characteristics of validity and reliability, able to provide useful measures patient-centered. A more complete detection of symptoms and their appropriate management may positively impact on the HRQoL of the patients and, indirectly, increase adherence to ART with expected positive impact on the efficacy of the treatment.

## Appendix

ISS-HIV symptoms scale. See Table 4.

**Table 4:** Have you suffered from any of the following symptoms over the past 4 weeks? A very great deal

		Not at all	A little	Moderately	A lot	A very great deal
1.	Tiredness	□	□	□	□	□
2.	Lack of appetite	□	□	□	□	□
3.	Weight loss	□	□	□	□	□
4.	Diarrhoea	□	□	□	□	□
5.	Heartburn	□	□	□	□	□
6.	Mental confusion	□	□	□	□	□
7.	Memory problems	□	□	□	□	□
8.	Sleep problems	□	□	□	□	□
9.	Impaired vision	□	□	□	□	□
10.	Coughing	□	□	□	□	□
11.	Shortness of breath	□	□	□	□	□
12.	Sweating	□	□	□	□	□
13.	Problems with sexual activity	□	□	□	□	□
14.	Decrease of sexual interest	□	□	□	□	□
15.	Abdominal bloating	□	□	□	□	□
16.	Abnormal accumulation of fat	□	□	□	□	□
17.	Headache	□	□	□	□	□
18.	Aching muscles	□	□	□	□	□
19.	Pain in the hands or feet	□	□	□	□	□
20.	Worrying	□	□	□	□	□
21.	Feeling sad	□	□	□	□	□
22.	Feeling anxious and nervous	□	□	□	□	□

## Abbrevations

PROs: patient reported outcomes
HRQoL: health related quality of life
ART: antiretroviral therapy
lSS-HIV symptoms scale: Istituto Superiore di Sanità-HIV symptoms scale
ISSQoL: Istituto Superiore di Sanità-Quality of Life
PCORI: Patient-Centered Outcomes Research Institute
EHR: electronic health records
EMR: electronic medical records

## References

[1] Food and Drug Administration (US). Federal Register. Guidance for Industry on Patient Reported Outcome Measures: Use in Medical Product Development to Support Labeling Claims.2009; Available at: https://www.federalregister.gov/articles/2009/12/09/. 10.1186/1477-7525-4-79 PMC1629006 17034633

[2] Webb A, Norton M (2004). Clinical assessment of symptom-focused health-related quality of life in HIV/AIDS. J Assoc Nurses AIDS Care.

[3] Detmer SB, Muller MJ, Schornagel JH, Wever LDV, Aaronson NK (2002). Health-related quality-of-life assessments and patient-physician communication: a randomized controlled trial. J Am Med Assoc.

[4] Wu AW (2000). Quality-of-life assessment comes of age in the era of highly active antiretroviral therapy. AIDS.

[5] Wu AW, Rubin HR (1992). Measuring health status and quality of life in HIV and AIDS. Psychol Health.

[6] Sanjay B, Chwastiak LA, Bruce RD (2005). Clinical management of depression and anxiety in HIV-infected adults. AIDS..

[7] Justice AC, Chang CH, Rabeneck L (2001). Clinical importance of provider-reported HIV symptoms compared with patient-report. Med Care..

[8] Fayer PM, Machin D.(2007).Quality of life. The assessment, analysis and interpretation of patient-reported outcomes, 2nd Edition: http://eu.wiley.com/WileyCDA/WileyTitle/productCd-047002450X.html. Accessed 10 Nov 2015.

[9] Garratt A, Schmidt L, Mackintosh A, Fitzpatrick R (2002). Quality of life measurement: bibliographic study of patient assessed health outcome measures. BMJ..

[10] Spilker B (1996). Quality of life and pharmaeconomics in clinical trials.

[11] Wu AW, Snyder CF, Clancy CM, Steinwachs DM (2010). Adding the patient perspective to comparative effectiveness research. Health Aff.

[12] Kroenke K, Price RK (1993). Symptoms in the community prevalence, classification, and psychiatric comorbidity. Arch Intern Me.

[13] Justice AC, Rabeneck L, Hays RD, Wu AW, Bozzette SA, For the Outcomes Committee of the AIDS Clinical Trials Group (1999). Sensitivity, specificity, reliability, and clinical validity of provider-reported symptoms: a comparison with self-reported symptoms. J Acquir Immune Defic Synd.

[14] Cunningham WE, Shapiro MF, Hays RD, Dixon WJ, Visscher BR, George WL (1998). Constitutional symptoms and health-related quality of life in patients with symptomatic HIV disease. Am J Med.

[15] Lorenz KA, Cunningham WE, Spritzer KL, Hays RD (2006). Changes in symptoms and health related quality of life in a nationally representative sample of adults in treatment for HIV. Qual Life Res.

[16] Lorenz KA, Shapiro MF, Asch SM, Bozzette SA, Hays RD (2001). Associations of symptoms and health-related quality of life: findings from a national study of persons with HIV infection. Ann Intern Med.

[17] Franchi D, Wenzel RP (1998). Measuring health-related quality of life among patients infected with human immunodeficiency virus. Clinical Infectious Dis.

[18] Haynes RB, Taylor DW, Sackett DL (1979). Compliance in health care.

[19] Barnhoorn F, Adriaanse H (1992). In search of factors responsible for noncompliance among tuberculosis patients in Wardha District. India Soc Sci Med.

[20] Ammassari A, Murri R, Pezzotti P, Trotta MP, Ravasio L, De Longis P (2001). Self-reported symptoms and medication side effects influence adherence to highly active antiretroviral therapy in patients with HIV infection. J Acquir Immune Defic Syndr.

[21] Grossman HA, Sullivan PS, Wu AW. Quality of life and HIV: current assessment tools and future directions for clinical practice. AIDS Read. 2003; 13(12): 583–590. http://www.medscape.com/viewarticle/467037_2 14959693

[22] Nunnaly JO (1978). Psycometric Theory.

[23] Bucciardini R, Murri R, Guarinieri M, Starace F, Martini M, Vatrella A (2006). ISSQoL: a new questionnaire for evaluating the quality of life of people living with HIV in the HAART era. Qual Life Res.

[24] Lauriola M, Murri R, Massella M, Mirra M, Donnini S, Fragola V (2010). A Factor Analytic Study of the Italian National Institute of Health Quality of Life—Core Evaluation Form (ISSQoL-CEF). Patient Prefer Adherence.

[25] Edelman EJ, Gordon K, Justice AC (2011). Patient and provider-reported symptoms in the Post-cART Era. AIDS Behav.

[26] Valderas JM, Kotzeva A, Espallargues M, Guyatt G, Ferrans CE, Halyard MY (2008). The impact of measuring patientreported outcomes in clinical practice: a systematic review of the literature. Qual Life Res.

[27] Boyce MB, Browne JP (2013). Does providing feedback on patient-reported outcomes to healthcare professionals result in better outcomes for patients? A systematic review. Qual Life Res.

[28] Marshall S, Haywood K, Fitzpatrick R (2006). Impact of patient-reported outcome measures on routine practice: a structured review. J Evaluation Clinical Pract.

[29] Greenhalgh J, Long AF, Flynn R (2005). The use of patient reported outcome measures in routine clinical practice: Lack of impact or lack of theory?. Soc Sci Med.

[30] Selby JV, Beal AC, Frank L (2012). The Patient-Centered Outcomes Research Institute (PCORI) National priorities for research and initial research agenda. JAMA.

[31] Wu AW. advances in the use of patient reported outcome measures in electronic health records. Including case Studies. Nov 7 2013. Available link: http://www.pcori.org/assets/2013/11/PCORI-PRO-Workshop-EHR-Landscape-Review-111913.pdf.

[32] Snyder CF, Jensen R, Courtin SO, Wu AW (2009). Patient viewpoint: a website for patient-reported outcomes assessment. Qual Life Res.

